# Effects of Resveratrol on Muscle Inflammation, Energy Utilisation, and Exercise Performance in an Eccentric Contraction Exercise Mouse Model

**DOI:** 10.3390/nu15010249

**Published:** 2023-01-03

**Authors:** Liang-Yu Su, Wen-Ching Huang, Nai-Wen Kan, Te-Hsuan Tung, Linh Ba Phuong Huynh, Shih-Yi Huang

**Affiliations:** 1 Graduate Institute of Metabolism and Obesity Sciences, Taipei Medical University, Taipei 110301, Taiwan; 2 Department of Exercise and Health Science, National Taipei University of Nursing and Health Sciences, Taipei 112303, Taiwan; 3 Office of Physical Education Affairs, Taipei Medical University Hospital, Taipei 110301, Taiwan; 4 School of Nutrition and Health Sciences, Taipei Medical University, Taipei 110301, Taiwan; 5 Department of Public Health, Nutrition and Food Safety, Lien Chieu Hospital, Danang 551000, Vietnam; 6 Nutrition Research Center, Taipei Medical University Hospital, Taipei 110301, Taiwan; 7 TMU Research Center for Digestive Medicine, Taipei Medical University, Taipei 110301, Taiwan

**Keywords:** resveratrol, eccentric contraction, downhill running, anti-inflammation, energy utilization

## Abstract

Eccentric contraction can easily cause muscle damage and an inflammatory response, which reduces the efficiency of muscle contraction. Resveratrol causes anti-inflammatory effects in muscles, accelerates muscle repair, and promotes exercise performance after contusion recovery. However, whether resveratrol provides the same benefits for sports injuries caused by eccentric contraction is unknown. Thus, we explored the effects of resveratrol on inflammation and energy metabolism. In this study, mice were divided into four groups: a control group, an exercise group (EX), an exercise with low-dose resveratrol group (EX + RES25), and an exercise with high-dose resveratrol group (EX + RES150). The results of an exhaustion test showed that the time before exhaustion of the EX + RES150 group was greater than that of the EX group. Tumour necrosis factor-α (*Tnfα*) mRNA expression was lower in the EX + RES150 group than in the EX group. The energy utilisation of the EX + RES150 group was greater than that of the EX + RES25 group in different muscles. High-dose resveratrol intervention decreased *Tnfα* mRNA expression and enhanced the mRNA expressions of sirtuin 1, glucose transporter 4, AMP-activated protein kinase α1, and AMP-activated protein kinase α2 in muscles. These results revealed that high-dose resveratrol supplementation can reduce inflammation and oxidation and improve energy utilisation during short-duration high-intensity exercise.

## 1. Introduction

A habit of exercise contributes to effective metabolism, strong muscles, and low fatigue [1] and protects against metabolic disorders and even cognitive impairment in older adults [2,3]. However, poor exercise habits can cause muscle damage. Mice were made to execute eccentric contraction that caused the overextension of muscle fibres [4]. When such muscle damage occurs, macrophages are polarised as M1 macrophages by tumour necrosis factor-α (TNF-α) and interleukin 6 (IL-6) to prevent damage to the muscle cells [4,5]. During a rest period, these M1 macrophages are converted to M2 macrophages by anti-inflammatory factors (i.e., insulin-like growth factor 1 and interleukin 10) for muscle reconstruction [5,6]. Increased levels of IL-6 and TNF-α in adipose tissue have been observed in individuals with obesity during long-duration eccentric contraction exercise [7]; however, the related cytokine mechanism is not understood.

For improved sports performance, 500 mg/d of resveratrol has been combined with physical exercise to improve mitochondrial volume density and muscle function and increase total myonuclei in older adults [8]. Resveratrol use has been reported to prevent muscle stiffness and soreness resulting from damage to sarcomeres in muscle fibre [9] and the excitation–contraction coupling system [10]; damage which further causes delayed onset muscle soreness. Studies have also supported that resveratrol is involved in the activation of AMP-activated protein kinase (AMPK) during muscle contraction over a longer exercise period [11]. Active AMPK is attributed to the translocation and activation of glucose transporter 4 (GLUT4) to improve glucose utilisation and the proliferation of mitochondria in muscles [12].

Most resting muscles present as contracted. However, in the early stages of muscle excitement, fewer reactive oxidative species (ROS), which promote more muscle contraction to balance energy utilisation, are produced [13]. The more intensive the exercise is, the more ROS are produced by muscle excitement [14]. During high-intensity short-duration exercise, greater muscle contraction causes more ROS production, resulting in injury and reduced muscle contraction as well as overtraining syndrome (OTS) [13]. Cheng et al. reported that selected antioxidant treatments did not improve force recovery after fatiguing stimulation of skeletal muscle fibres in a mouse model [15]. However, some studies have shown that intervention targeting selected antioxidants altered oxidative stress in athletes with OTS by reducing plasma malondialdehyde levels [16] and resulted in improved muscle function in individuals with adjuvant-induced arthritis [17]. The effects of antioxidant intervention in various exercise types remain disputed and unclear.

Resveratrol use has been reported to benefit health and has therapeutic effects in humans [18]. Many studies have shown that resveratrol possesses antiaging [19], anticancer, anti-atherosclerosis, and anti-inflammatory effects; increases insulin sensitivity; and contributes to the reduction in ROS levels [20]. Resveratrol presents as a *trans*-form polyphenolic compound in nature, commonly found in grape skin, cherries, and peanuts. When consumed orally, trans-resveratrol is rapidly converted to the more biologically active form of dihydroresveratrol [21]. This resveratrol form has been reported to regulate sirtuin 1 (SIRT 1), peroxisome proliferator-activated receptor gamma coactivator 1-α (PGC-1α), AMPK, and TNF-α, which enforces cell mitochondrial function, increases insulin sensitivity, and inhibits inflammation and low-density lipoprotein oxidation in individuals with diabetes [20,22,23].

This study used a downhill running exercise to mimic eccentric contraction injury and assess the efficacy of resveratrol in mice models. We hypothesised that giving mice different doses of resveratrol would protect against the damage caused by short-duration high-intensity eccentric exercise. The mechanisms of resveratrol involved in muscle inflammation, energy utilisation, and exercise performance were also evaluated.

## 2. Materials and Methods

### 2.1. Materials

Trans-resveratrol was provided by Tokyo Chemistry Industry. All chemicals used in this study were obtained from Sigma-Aldrich (St. Louis, MO, USA).

### 2.2. Animals

C57BL/6J mice (aged 6 weeks) were purchased from the National Laboratory Animal Center (Taipei, Taiwan). All animals were fed on a chow diet (Rodent Laboratory Chow 5001, LabDiet, St. Louis, MO, USA) and distilled water *ad libitum*. Mice were housed in a regular cycle (12 h light/dark) at room temperature (23 ± 2 °C) and 60–70% humidity in the Taipei Medical University Laboratory Animal Center. All animal experimental protocols were reviewed and approved by the Institutional Animal Care and Use Committee of Taipei Medical University (LAC-2021-0310).

### 2.3. Experiment Design

**Figure 1:**
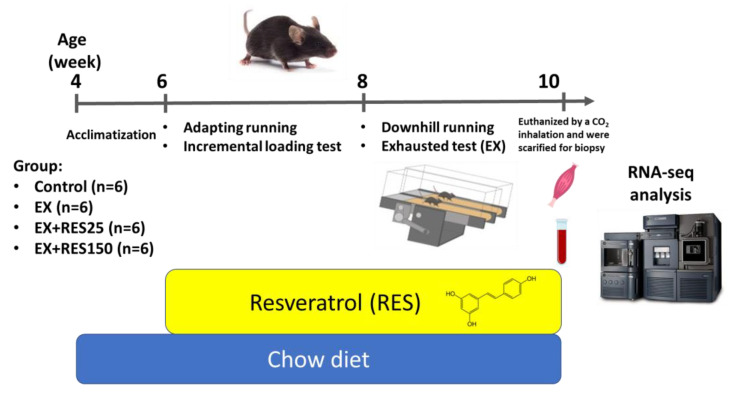
Experimental design of short-term downhill training. NC: control (*n* = 6); EX: exercise (*n* = 6); EX + RES25: exercise with resveratrol 25 mg/kg (*n* = 6); EX + RES150: exercise with resveratrol 150 mg/kg (*n* = 6).

C57BL/6J mice were housed in the animal facility for 1 week for them to adapt to the environment before the study. Twenty-four mice were randomly assigned into four equal groups: control (NC), exercise (EX), exercise with low-dose resveratrol (25 mg/kg body weight; EX + RES25), and exercise with high-dose resveratrol (150 mg/kg body weight; EX + RES150). The mice in the EX, EX + RES25, and EX + RES150 groups underwent 3 days of acclimation running before undertaking the incremental load test (ILT). Acclimation involved running 10 m/min for 15 min with an incline of 0° each day for 3 days. All the mice underwent an exhaustion test the day after the ILT, and the downhill running section of the study started the following week. Resveratrol was dissolved in distilled water. EX-treated mice were gavaged with various amounts (25 mg/kg/d [RES25] and 150 mg/kg/d [RES150] in 0.5 mL of normal saline) of resveratrol or the vehicle (0.5 mL of normal saline) for C and EX groups for 4 weeks (Figure 1).

### 2.4. ILT, Exhaustion Test, and Downhill Running

Mice undertook the ILT on a motorised treadmill (Rodent Treadmill 47300, Ugo Basile, Italy). The intensity of exercise was programmed to increase by 3 m/min (initial speed: 12 m/min) in the first 3 min and mice ran on a 0% gradient until exhaustion [24]. Individual exhaustion velocity (EV) was determined from the results. The same exercise protocol was used in the exhaustion test. After a 3-day acclimation to running and the ILT, the mice of groups EX, EX + RES25 and EX + RES150 ran on a motorised treadmill (−15° slope) at 22 m/min (60% EV), 2 h/day, 5 days/week for 2 weeks [25]. Training interventions were conducted between 10 am and 6 pm.

### 2.5. Sample Collection

After the downhill running test, mice were euthanized by a CO_2_ inhalation overdose and were sacrificed for biopsy, including biopsies of the liver, epididymal adipose tissue (eWAT), gastrocnemius muscle, soleus muscle, and tibialis anterior muscle, which were collected and stored at −80 °C for biochemical analyses. Skin irritation was also evaluated in all animals at the end of the study.

### 2.6. Quantitative Reverse Transcription Polymerase Chain Reaction (RT-qPCR)

RNA was extracted using RNAzol RT (Molecular Research Center, Cincinnati, OH, USA) according to manufacturer guidelines. 1 μg of RNA was reverse transcribed using a TOOLSQuant II fast RT kit (BIOTOOLS, Taiwan). Genes were quantified using specific primers with SYBR green using the Applied Biosystems QuantStudio 1 Real-Time PCR System. The final quantification was executed using the 2−∆∆CT method with glyceraldehyde 3-phosphate dehydrogenase (GAPDH) primer as a control. The sequences of primers are shown in Table 1.

### 2.7. Next Generation Sequencing Analysis

First, cDNA libraries were collected from four independent samples in each group. The cDNA libraries were assessed using the Agilent 2100 Bioanalyzer system and the Real-Time PCR system. β-Actin served as an internal control to verify the quality and quantity of cDNA in the PCR system. The sequenced libraries underwent fragment size detection using the Agilent Bioanalyzer 2100 system and library concentration determination using the Real-Time PCR system; the quality-confirmed libraries underwent 150-bp paired-end sequencing on the Illumina NovaSeq 6000 sequencer (Genomics, BioSci & Tech Company, New Taipei City, Taiwan). Raw sequencing reads were filtered using Trimmomatic (version 0.36) [26]. Reads were aligned using Bowtie2 (version 2.3.5) [27]. Raw gene counts were extracted using RSEM (version 1.3.3) [28]. The R package edgeR (v3.16.5) was used for the differential gene expression analysis of two sample groups. Furthermore, the log_2_ fold change was calculated as log_2_(sample count 1/sample count 2). The *t*-test was used to identify significant differences between samples from the two groups (*p* < 0.05). Gene ontology (GO) enrichment analysis was conducted on the differential genes obtained through screening. When *p* < 0.05, GO terms were significantly enriched [29]. The Kyoto Encyclopedia of Genes and Genomes was used for the gene enrichment of differentially expressed genes.

### 2.8. Statistical Analysis

All results are expressed in terms of the mean ± SEM. The significance of the difference was examined using GraphPad Prism version 9.0 (GraphPad Software; San Diego, CA, USA). All data were normally distributed. Significant differences were analyzed using a student’s t-test or one-way analysis of variance with Tukey’s test and Bonferroni’s test for multiple comparisons. A *p*-value less than 0.05 was considered statistically significant.

## 3. Results

### 3.1. Average Body Weight, Food Intake, and Tissue Relative Weight

Average body weight, food intake, and tissue relative weight are shown in Table 2. No significant differences in the body weight, food and water intake, and selected biopsy results were found. However, the relative weights of the eWAT in the EX, EX + RES25, and EX + RES150 groups were significantly lower than that of the NC group (*p* < 0.05).

### 3.2. Exercise Performance in Exhaustion Test

**Figure 2:**
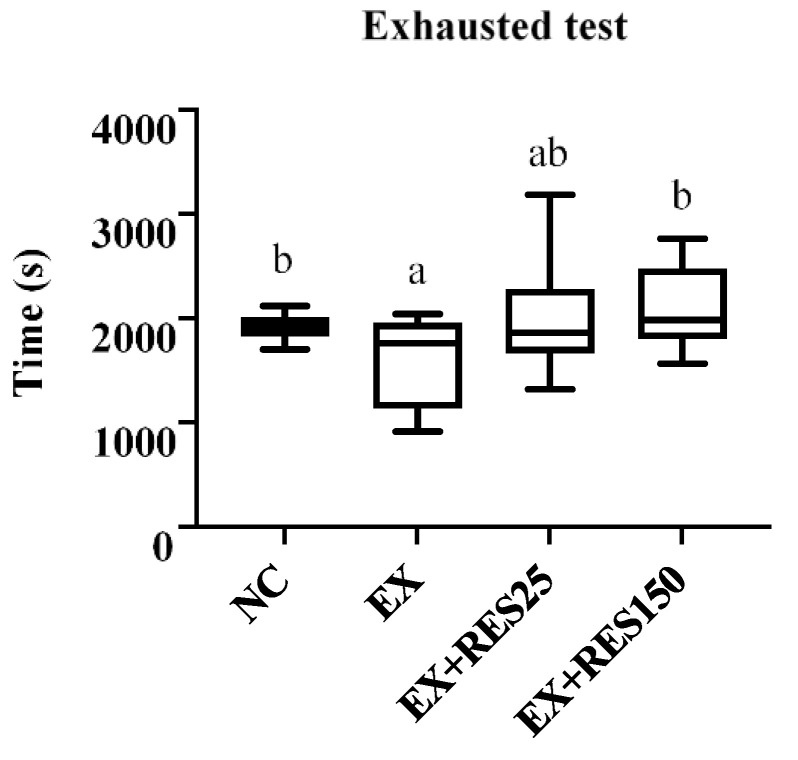
Exhaustion test in different groups. NC: control (*n* = 6); EX: exercise (*n* = 6); EX + RES25: exercise with resveratrol 25 mg/kg (*n* = 6); EX + RES150: exercise with resveratrol 150 mg/kg (*n* = 6). Data are presented as mean ± SEM. Data were analysed using one-way analysis of variance and a *t*-test followed by the Bonferroni multiple comparison test. Superscript letters (a and b) in columns denote a significant difference (*p* < 0.05).

The time before exhaustion in the EX group was shorter than in the NC group (*p* < 0.05); however, the time before exhaustion of the EX and EX + RES25 groups showed no difference. A significant difference was found between the results of the EX and EX + RES150 groups (*p* < 0.05; Figure 2).

### 3.3. Lactate Dehydrogenase (LDH) and Creatine Kinase (CK)

**Figure 3:**
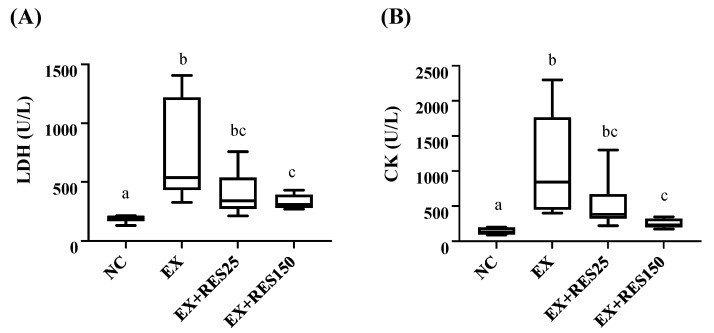
Effects of resveratrol on (**A**) lactate dehydrogenase and (**B**) creatine kinase with eccentric exercise–induced muscle damage. NC: control (*n* = 6); EX: exercise (*n* = 6); EX + RES25: exercise with resveratrol 25 mg/kg (*n* = 6); EX + RES150: exercise with resveratrol 150 mg/kg (*n* = 6). Data are presented as mean ± SEM. Data were analysed using one-way analysis of variance and a t-test followed by the Bonferroni multiple comparison test. Superscript letters (a, b and c) in columns denote a significant difference (*p* < 0.05).

The blood biochemical values of the mice after the exhaustion test showed that the levels of LDH and CK in the EX group were significantly higher than those in the NC group (*p* < 0.05). Compared with the NC group, LDH and CK in the EX + RES25 and EX + RES150 groups were also significantly increased (*p* < 0.05 for both), but LDH and CK in the EX + RES150 group were significantly lower than those in the EX group (*p* < 0.05; Figure 3).

### 3.4. Gene Expression of Inflammatory Factors in Muscles

#### 3.4.1. Gastrocnemius Muscle

**Figure 4:**
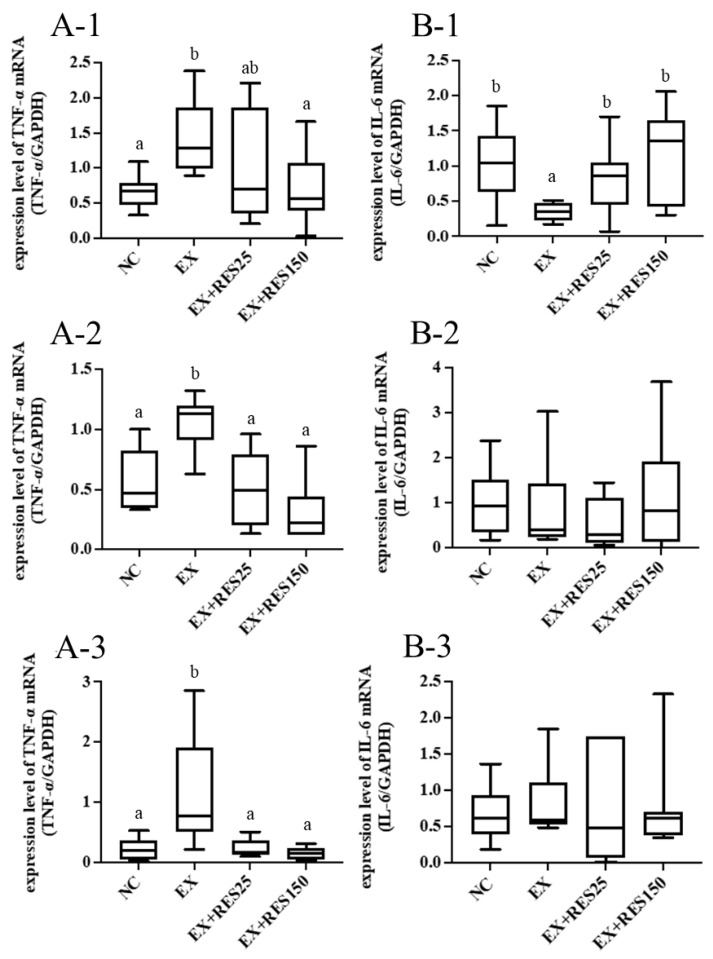
Effects of resveratrol on inflammatory cytokines (**A**) *Tnfα* and (**B**) IL-6 mRNA expression in (1) gastrocnemius muscle, (2) tibialis anterior muscle, and (3) soleus muscle during short-duration downhill running. mRNA expression was normalized to GAPDH and expressed as fold change relative to the control group. NC: control (*n* = 6); EX: exercise (*n* = 6); EX + RES25: exercise with resveratrol 25 mg/kg (*n* = 6); EX + RES150: exercise with resveratrol 150 mg/kg (*n* = 6). Data are presented as mean ± SEM. Data were analysed using one-way analysis of variance and a t-test followed by the Bonferroni multiple comparison test. Superscript letters (a and b) in columns denote a significant difference (*p* < 0.05).

Compared with that of the NC group and the EX + RES150 group, the mRNA expression of TNF-α in the gastrocnemius muscle was increased higher in the EX group (*p* < 0.05; Figure 4A-1). *Il6* mRNA expression was significantly lower in the EX group than in the other groups (*p* < 0.05; Figure 4B-1).

#### 3.4.2. Tibialis Anterior Muscle

Compared with the NC group, the mRNA expression of TNF-α and IL-6 in the tibialis anterior muscle were significantly higher in the EX, EX + RES25, and EX + RES150 groups (*p* < 0.05; Figure 4A-2,B-2). *Tnfα* mRNA expression in the EX + RES25 and EX + RES150 groups was significantly higher than that in the NC group (*p* < 0.05; Figure 4A-2). The mRNA expression of TNF-α in the EX + RES25 and EX + RES150 groups was significantly lower than that in the EX group (Figure 4A-2). No difference in *Il6* mRNA expression among the four groups was found (Figure 4B-2).

#### 3.4.3. Soleus Muscle

Compared with that of the NC group, the mRNA expression of TNF-α in the soleus muscle was significantly higher in the EX group. After resveratrol was administered, soleus-muscle *Tnfα* mRNA expression in the EX + RES25 and EX + RES150 groups was significantly lower than that in the EX group (*p* < 0.05; Figure 4A-3). No difference in soleus muscle *Il6* mRNA expression between the groups was found (Figure 4B-3).

### 3.5. Gene Expression of Energy Metabolism and Antioxidant Factors in Muscles

#### 3.5.1. Gastrocnemius Muscle

**Figure 5:**
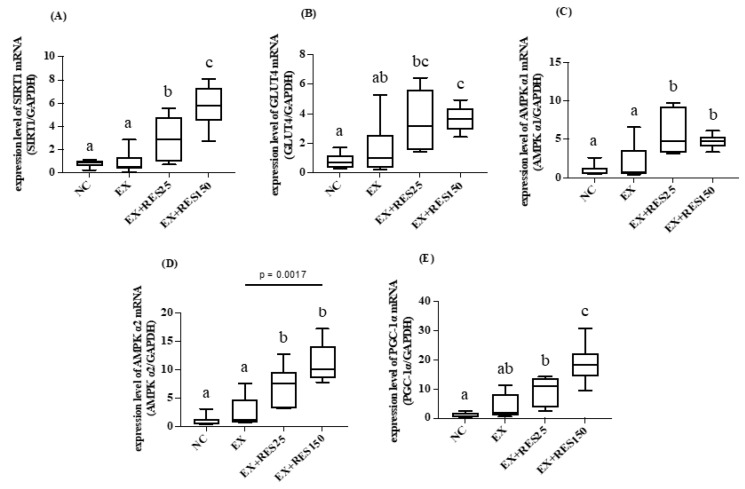
Effects of resveratrol on energy utilization and antioxidative mRNA expression in gastrocnemius muscle during short-duration downhill running. (**A**) *Sirt1* mRNA (**B**) *Ampkα1* mRNA (**C**) *Ampkα2* mRNA (**D**) *Glut4* mRNA (**E**) *Pgc1α* mRNA. mRNA expression was normalized to GAPDH and is expressed in terms of fold change relative to the control group. NC: control (*n* = 6); EX: exercise (*n* = 6); EX + RES25: exercise with resveratrol 25 mg/kg (*n* = 6); EX + RES150: exercise with resveratrol 150 mg/kg (*n* = 6). Data are presented as mean ± SEM. Data were analysed using one-way analysis of variance and a t-test followed by the Bonferroni multiple comparison test. Superscript letters (a, b and c) in columns denote a significant difference (*p* < 0.05).

The results showed that the mRNA expression of SIRT1, AMPK α1, and AMPK α2 in the gastrocnemius muscle in the EX + RES25 group was significantly higher than that in the EX group (*p* < 0.05; Figure 5A,C,D). The mRNA expression levels of GLUT4, AMPK α1, and PGC-1α in the gastrocnemius muscle in the EX + RES150 group were significantly higher than those in the EX group (*p* < 0.05; Figure 5B,C,E). The mRNA expression of SIRT1 and PGC-1α significantly differed between the EX + RES25 and EX + RES150 groups (*p* < 0.05; Figure 5A,E).

#### 3.5.2. Tibialis Anterior Muscle

**Figure 6:**
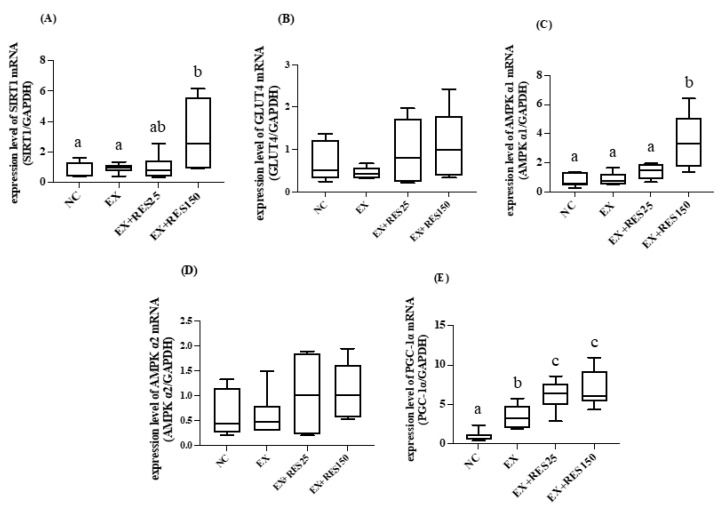
Effects of resveratrol on energy utilization and antioxidative mRNA expression in tibialis anterior muscle during short-duration downhill running training. (**A**) *Sirt1* mRNA (**B**) *Ampkα1* mRNA (**C**) *Ampkα2* mRNA (**D**) *Glut4* mRNA (**E**) *Pgc1α* mRNA. mRNA expression was normalized to GAPDH and is expressed in terms of fold change relative to the control group. NC: control (*n* = 6); EX: exercise (*n* = 6); EX + RES25: exercise with resveratrol 25 mg/kg (*n* = 6); EX + RES150: exercise with resveratrol 150 mg/kg (*n* = 6). Data are presented as mean ± SEM. Data were analysed using one-way analysis of variance and a t-test followed by the Bonferroni multiple comparison test. Superscript letters (a, b and c) in columns denote a significant difference (*p* < 0.05).

The mRNA expression levels of SIRT1 and AMPK α1 in the tibialis anterior muscle in the EX + RES150 group were significantly higher compared with those in the NC and EX groups (*p* < 0.05; Figure 6A,C). The mRNA expression of AMPK α1 increased significantly with resveratrol dosage, exhibiting a dose-dependent effect in the two RES groups (*p* < 0.05; Figure 6C). The mRNA expression of PGC-1α was significantly higher in the EX, EX + RES25, and EX + RES150 groups than in the NC group (*p* < 0.05; Figure 6E). No difference between the groups in the expression of GLUT4 and AMPK α2 was found (Figure 6B,D).

#### 3.5.3. Soleus Muscle

**Figure 7:**
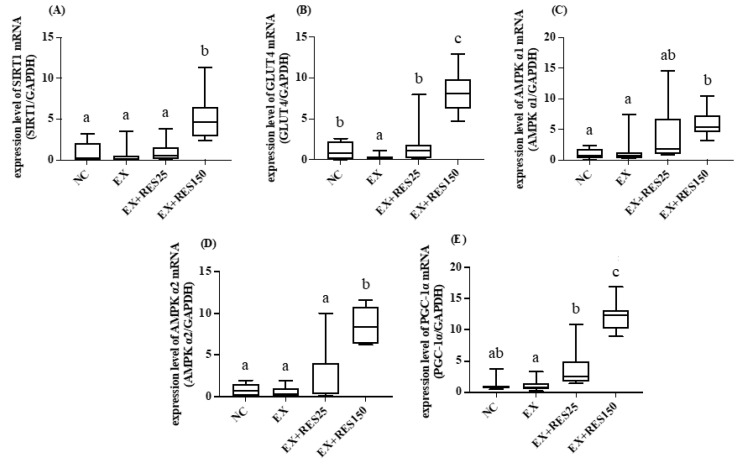
Effects of resveratrol on energy utilization and antioxidative mRNA expression in soleus muscle during short-duration downhill running training. (**A**) *Sirt1* mRNA (**B**) *Ampkα1* mRNA (**C**) *Ampα2* mRNA (**D**) *Glut4* mRNA (**E**) *Pgc1α* mRNA. mRNA expression was normalized to GAPDH and is expressed in terms of fold change relative to the control group. NC: control (*n* = 6); EX: exercise (*n* = 6); EX + RES25: exercise with resveratrol 25 mg/kg (*n* = 6); EX + RES150: exercise with resveratrol 150 mg/kg (*n* = 6). Data are presented as mean ± SEM. Data were analysed using one-way analysis of variance and a t-test followed by the Bonferroni multiple comparison test. Superscript letters (a, b and c) in columns denote a significant difference (*p* < 0.05).

The soleus muscle mRNA expression levels of SIRT1, GLUT4, AMPK α1, AMPK α2, and PGC-1α in the EX + RES150 group were significantly higher than in the NC group (*p* < 0.05; Figure 6). *Sirt1*, *Glut4*, *Ampkα2*, and *Pgc1α* mRNA expression increased with resveratrol dosage significantly (*p* < 0.05; Figure 7A,B,D,E). The mRNA expression level of GLUT4 in the EX group was significantly lower than in the NC group (Figure 7B).

### 3.6. MA Plot, Volcano Plot, and Bar Charts for EX vs. EX + RES150 Groups

**Figure 8:**
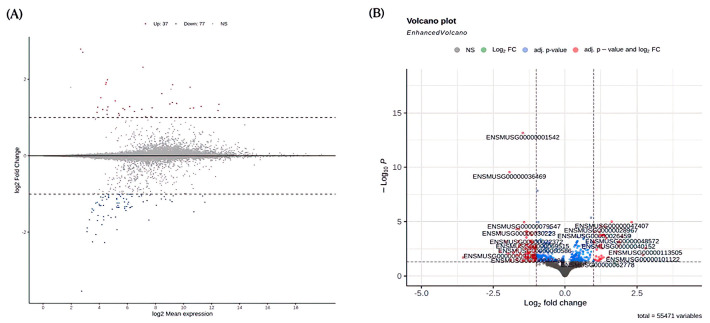
Different gene expression in EX + RES150 and EX groups. (**A**) MA plot illustrating the distribution of upregulated and downregulated genes (coloured dots) for EX + RES150 group vs. EX group; (**B**) volcano plot illustrating significance and fold change of the up and downregulated genes (coloured dots) for EX + RES150 group vs. EX group.

In the EX and EX + RES150 groups, 37 genes were more significantly upregulated, and 77 genes were more significantly downregulated in the high-dose group than in the EX group (Figure 8).

**Figure 9:**
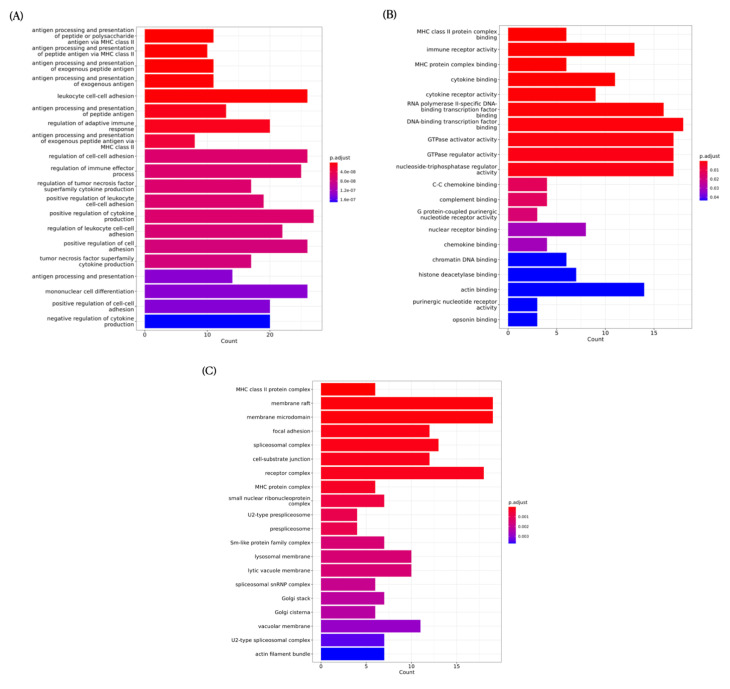
Gene ontology (GO) analysis of EX + RES150 group vs. NC group. (**A**) GO analysis of biological process pathways for EX + RES150 group vs. EX group; (**B**) GO analysis of molecular function pathways for EX + RES150 group vs. EX group; (**C**) GO analysis of cellular component pathways for EX + RES150 group vs. EX group.

As shown in the biological process plot, pathway regulation occurred due to the intervention of high-dose resveratrol, such as the positive regulation of cytokine production (Figure 9A). Analysis of molecular function showed that the regulation of pathways related to energy hydrolysis enzymes also occurred, indicated, for example, by nucleoside-triphosphatase regulator activity and GTPase regulator activity (Figure 9B). In addition, as shown by the cellular component bar chart, most regulated pathways were found to be related to immunity, such as MHC class II proteins or lysosomal membrane proteins (Figure 9C). Higher doses of resveratrol have a greater effect on facial features during exercise and may have greater anti-inflammatory, energy utilisation-related, and immune-boosting effects.

## 4. Discussion

In this experiment, we used short-duration downhill running and different dosages of resveratrol to evaluate inflammation and energy utilisation in the different muscles of mice. The main findings were: (1) the time before exhaustion decreased due to exercise injury; this was counteracted by the intervention of resveratrol; (2) inflammation in the muscles was reduced by the intervention of resveratrol; (3) the expression of genes related to energy utilisation in the EX + RES150 group was significantly higher than in the EX group, indicating that the intervention of resveratrol increases muscle energy utilisation.

The time to exhaustion in the EX group was significantly lower than that in the NC, EX + RES25, and EX + RES150 groups. The results of this experiment demonstrate that resveratrol supplementation is beneficial for endurance exercise. Our study found no difference in endurance performance between the low-dose group and the high-dose group. This difference between this study and ours may have been due to differences in exercise mode (weight-loaded swimming in the previous study).

TNF-α and IL-6 levels are often used to indicate inflammation. When an inflammatory response occurs, the production of cytokines in injured tissues increases significantly. *Tnfα* mRNA expression in the EX group was significantly higher than that in the NC group, confirming that exercise injury elevated the production of TNF-α [30]. Resveratrol effectively reduced inflammation; high doses of resveratrol exerted anti-inflammatory effects in different muscles. In long-duration exercises, reaching exhaustion always results in high levels of oxidative stress. A high-intensity cycling-based clinical trial demonstrated that resveratrol supplementation attenuated exercise-induced serum interleukin-6 levels but not oxidative stress [31]. Such physical phenomena imply that resveratrol affects anti-inflammation and antioxidation processes independently. IL-6 production is positively correlated with glucose uptake by GLUT4 in muscles [32,33,34]. In our study, the mRNA expression of IL-6 did not differ between different muscle tissues, but the repression of *Glut4* mRNA was observed in the gastrocnemius and soleus muscles. This suggests that increased mRNA levels of IL-6 and GLUT4 occur synchronously; thus, IL-6 may trigger GLUT4-induced glucose utilisation in muscle.

Many studies have found that during endurance exercise, due to the reduction in available glycogen in the body, more AMPK is activated to facilitate the production of downstream products, providing the body with energy [35]. During exercise, the intervention of resveratrol resulted in the activation not only of AMPK but also SIRT1 and its downstream proteins GLUT4 and PGC-1α and in miR-22-3p in a muscle cell model [36]. Resveratrol also exhibited dual bioactive effects in our study; however, the effects of resveratrol remain unclear.

Type I muscle fibres support long-duration exercise, type II fibres support explosive short-duration exercise, and type IIx fibres transform into different types of fibres in relation to the intensity of the physical activity at hand [37]. The mRNA expression of AMPK α1 in the gastrocnemius and tibialis anterior muscles in the EX group did not differ from that in the NC group. We hypothesise that the type IIx muscle fibres in these muscles were not converted into type I muscle fibres because the exercise period was not long enough for the fibres to adapt to endurance exercise. The effect of resveratrol on AMPK activation in the gastrocnemius and tibialis anterior muscles was greater than that caused by exercise. However, no differences in *Ampkα1* and *Ampkα2* mRNA expression in the soleus muscle between the EX and the NC groups were found. These apparently conflicting results were also observed in a 12-week study [38], and we speculate that the two-week duration of this experiment was insufficient for a long-term exercise study. However, the soleus muscle has a high proportion of red muscle, which supports aerobic exercise. Thus, the significant differences between the NC and EX groups can be attributed to resveratrol intervention.

The mRNA expression of GLUT4 in the gastrocnemius muscle and soleus muscle in the EX + RES150 group was significantly higher than that in the EX group. This result is consistent with the findings of a previous study that the intervention of resveratrol in muscle cells effectively increases the amount of GLUT4 translocated to the plasma membrane [39]. The tibialis anterior muscle does not exhibit similar effects; the ratio of red to white muscle in the tibialis anterior muscle may contribute to this [38]. Studies have demonstrated that white muscle fibres can activate GLUT4 translocation during exercise [40]; the gastrocnemius muscle has a higher proportion of white muscle and exhibits higher *Glut4* mRNA expression. Pereira BC et al. reported that excessive eccentric contraction training impairs insulin signal transduction in mice’s skeletal muscles and reduces the rate of GLUT4 translocation [41]. Thus, muscle injury may have caused the mRNA expression of GLUT4 in the soleus muscle in the EX group to be significantly lower than in the other groups.

As an oxidoreductase, SIRT1 regulates various cellular events, including apoptosis, cell survival, endocrine signalling, and gene transcription [42]. SIRT1 activates the downstream product PGC-1α, and PGC-1α regulates mitochondrial biosynthesis to facilitate the regulation of oxidative metabolism [43]. The mRNA expression of SIRT1 and PGC-1α in the gastrocnemius muscle, tibialis anterior muscle, and soleus muscle increased significantly with the intervention of high-dose resveratrol compared with the NC group, which is consistent with the results of previous studies [44], indicating resveratrol intervention for short-duration exercise can effectively increase energy utilisation and cause an antioxidising effect.

In this study, we assessed genome expression under resveratrol intervention in short-duration eccentric contraction exercises. We focused on muscle inflammation and energy utilisation–related gene expression. One gene that was intensively activated after high-dose resveratrol intervention was myosin light chain kinase 2 (ENSMUSG00000027470), which is intensively expressed in cardiac and skeletal muscle [45] and regulates the interaction of myosin and actin through the concentration of calcium ions protein cross bridge [46]. In this experiment, high-dose resveratrol intensively activated this gene, thus we hypothesise that resveratrol increases muscle contraction ability during exercise. NADH dehydrogenase subunit 5 (ENSMUSG00000064367) is part of mitochondrial complex I, a large enzyme complex that is active in mitochondria [47]. We found that this gene was upregulated in the presence of intervening resveratrol. Therefore, we hypothesise that due to the increase in the expression of this gene, oxidative phosphorylation on the inner mitochondrial membrane and the production of ATP increased, providing the cell with more energy and thereby increasing the utilisation of energy during exercise. Gene ontology enrichment table of EX vs. EX + RES150 included biological process, molec-ular function and cellular component is shown in Supplementary Table S1.

## 5. Conclusions

High-dose resveratrol intervention prolonged the time before exhaustion for short-duration downhill running. High-dose resveratrol intervention decreased *Tnfα* mRNA expression and enhanced the mRNA expressions of SIRT1, GLUT4, AMPK α1, and AMPK α2 in some muscles. These results indicate that high-dose resveratrol supplementation can reduce inflammation and oxidation and improve the utilisation of energy during short-duration high-intensity exercise.

## Figures and Tables

**Table 1:** Primers list for qPCR.

Genes	Direction	Primer Sequence (5′to 3′)
*Gapdh*	Forward	TGGTGAAGGTCGGTGTGAAC
	Reverse	AATGAAGGGGTCGTTGATGG
*Tnfα*	Forward	CCACCACGCTCTTCTGTCTAC
	Reverse	AGGGTCTGGGCCATAGAACT
*Il6*	Forward	GCTTAATTACACATGTTCTCTGGGAAA
	Reverse	CAAGTGCATCATCGTTGTTCATAC
*Il1β*	Forward	TGGACCTTCCAGGATGAGGACA
	Reverse	GTTCATCTCGGAGCCTGTAGTG
*Sirt1*	Forward	CAGACCCTCAAGCCATGTTT
	Reverse	ACACAGAGACGGCTGGAACT
*Glut4*	Forward	GTAACTTCATTGTCGGCATGG
	Reverse	AGCTGAGATCTGGTCAAACG
*Ampkα1*	Forward	CTCAGTTCCTGGAGAAAGATGG
	Reverse	CTGCCGGTTGAGTATCTTCAC
*Ampkα2*	Forward	CAGGCCATAAAGTGGCAGTTA
	Reverse	AAAAGTCTGTCGGAGTGCTGA
*Pgc1α*	Forward	TGATGTGAATGACTTGGATACAGACA
	Reverse	GCTCATTGTTGTACTGGTTGGATATG

**Table 2:** Body weight, food and water intake and tissue relative weight in mice.

Parameters	NC	EX	EX + RES25	EX + RES150
Body weight (g)	23.2 ± 0.5	23.5 ± 0.5	23.3 ± 0.4	23.3 ± 0.5
Food intake (g/mice/day)	6.10 ± 0.71	6.60 ± 0.59	6.60 ± 0.61	6.50 ± 0.81
Water intake (mL/mice/day)	3.80 ± 0.06	4.00 ± 0.03	4.00 ± 0.03	4.00 ± 0.09
Liver (% of BW)	3.80 ± 0.05	3.80 ± 0.22	3.50 ± 0.07	3.50 ± 0.08
Gastrocnemius muscle (% of BW)	1.10 ± 0.03	1.10 ± 0.03	1.10 ± 0.02	1.10 ± 0.02
Tibialis anterior muscle (% of BW)	0.36 ± 0.03	0.36 ± 0.03	0.34 ± 0.02	0.37 ± 0.03
eWAT (% of BW)	1.00 ± 0.05 ^a^	0.61 ± 0.05 ^b^	0.60 ± 0.03 ^b^	0.68 ± 0.04 ^b^

Data displayed as mean ± S.E.M. Data were analysed using one-way analysis of variance and a *t*-test followed by the Bonferroni multiple comparison test. Different superscripts (a and b) in each row indicate significant differences among groups (*p* < 0.05).

## Data Availability

Not applicable.

## Supplementary Materials

The following supporting information can be downloaded at: https://www.mdpi.com/article/10.3390/nu15010249/s1, Table S1: Gene ontology enrichment table of EX vs. EX+RES150—biological process, molecular function & cellular component.

## Abbreviations

AMPK: AMP activated protein kinase; CK, creatine kinase; COX-2, cyclooxygenase-2; DOMS, delayed onset muscle soreness; EC, excitation-contraction; EV, exhaustion velocity; FOXO1, forkhead box O1; GSH, glutathione; GSH-PX, glutathione peroxidase; GSSG, oxidized glutathione; HIIT, high intensity interval training; IGF-1, insulin-like growth factor-1; IL-1, interleukin-1; IL-6, interleukin-6; IL-10, interleukin-10, LDH, lactate dehydrogenase; MDA, malondialdehyde, NFAT, nuclear factor of activated T cells; NF-κB, nuclear factor kappa-light-chain-enhancer of activated B cells; PGC-1α, peroxisome proliferator-activated receptor gamma coactivator 1-alpha; ROS, reactive oxygen species; TBARS, 2-thiobarbituric acid reacting substances test; TNF-α, tumor necrosis factor- α; SIRT 1, sirtuin 1.
