# Supplementary material for: Effects of Resveratrol on Muscle Inflammation, Energy Utilisation, and Exercise Performance in an Eccentric Contraction Exercise Mouse Model
Source: Nutrients. 2023 Jan 3;15(1):249. doi: 10.3390/nu15010249 (PMC9824440; doi:10.3390/nu15010249)
Supplement: Supplementary file 1 [file nutrients-15-00249-s001.zip › nutrients-2115054-supplementary.pdf]

Table S1. Gene ontology enrichment table of EX vs. EX+RES150 – biological process, molecular function & cellular component

(A) Gene Ontology enrichment table - Biological Process (BP)

ID	Description	Count	GcncRatio	BgRatio	pvalue	qvalue
GO:0002504	antigen processing and presentation of peptide or polysaccharide antigen via MHC class II	11	11/360	26/29008	0	0e+00
GO:0002495	antigen processing and presentation of peptide antigen via MHC class II	10	10/360	24/29008	0	0e+00
GO:0002478	antigen processing and presentation of exogenous peptide antigen	11	11/360	33/29008	0	0e+00
GO:0019884	antigen processing and presentation of exogenous antigen	11	11/360	40/29008	0	0e+00
GO:0007159	leukocyte cell-cell adhesion	26	26/360	373/29008	0	0e+00
GO:0048002	antigen processing and presentation of peptide antigen	13	13/360	78/29008	0	0e+00
GO:0002819	regulation of adaptive immune response	20	20/360	236/29008	0	0e+00
GO:0019886	antigen processing and presentation of exogenous peptide antigen via MHC class II	8	8/360	20/29008	0	0e+00
GO:0022407	regulation of cell-cell adhesion	26	26/360	457/29008	0	0e+00
GO:0002697	regulation of immune effector process	25	25/360	424/29008	0	0e+00
GO:1903555	regulation of tumor necrosis factor superfamily cytokine production	17	17/360	186/29008	0	0e+00
GO:1903039	positive regulation of leukocyte cell-cell adhesion	19	19/360	240/29008	0	0e+00

GO:0001819	positive regulation of cytokine production	27	27/360	500/29008	0	0e+00
GO:1903037	regulation of leukocyte cell-cell adhesion	22	22/360	333/29008	0	0e+00
GO:0045785	positive regulation of cell adhesion	26	26/360	471/29008	0	1e-07
GO:0071706	tumor necrosis factor superfamily cytokine production	17	17/360	191/29008	0	le-07
GO:0019882	antigen processing and presentation	14	14/360	126/29008	0	le-07
GO:1903131	mononuclear cell differentiation	26	26/360	489/29008	0	le-07
GO:0022409	positive regulation of cell-cell adhesion	20	20/360	288/29008	0	le-07
GO:0001818	negative regulation of cytokine production	20	20/360	292/29008	0	le-07

(B) Gene Ontology enrichment table - Molecular Function (MF)

ID	Description	Count	GeneRatio	BgRatio	pvalue	qvalue
GO:0023026	MHC class II protein complex binding	6	6/355	13/28438	0.0000000	0.0000026
GO:0140375	immune receptor activity	13	13/355	136/28438	0.0000000	0.0000039
GO:0023023	MHC protein complex binding	6	6/355	23/28438	0.0000003	0.0000460
GO:0019955	cytokine binding	11	11/355	139/28438	0.0000015	0.0001666
GO:0004896	cytokine receptor activity	9	9/355	97/28438	0.0000036	0.0003263
GO:0061629	RNA polymerase Il-specific DNA-binding transcription factor binding	16	16/355	329/28438	0.0000045	0.0003401
GO:0140297	DNA-binding transcription factor binding	18	18/355	431/28438	0.0000095	0.0006136
GO:0005096	GTPase activator activity	17	17/355	405/28438	0.0000158	0.0008888

GO:0030695	GTPase regulator activity	17	17/355	421/28438	0.0000258	0.0011629
GO:0060589	nucleoside-triphosphatase regulator activity	17	17/355	421/28438	0.0000258	0.0011629
GO:0019957	C-C chemokine binding	4	4/355	25/28438	0.0002455	0.0100532
GO:0001848	complement binding	4	4/355	26/28438	0.0002872	0.0107843
GO:0045028	G protein-coupled purinergic nucleotide receptor activity	3	3/355	12/28438	0.0003903	0.0135264
GO:0016922	nuclear receptor binding	8	8/355	155/28438	0.0007649	0.0246156
GO:0019956	chemokine binding	4	4/355	34/28438	0.0008237	0.0247403
GO:0031490	chromatin DNA binding	6	6/355	97/28438	0.0013818	0.0345668
GO:0042826	histone deacetylase binding	7	7/355	132/28438	0.0013843	0.0345668
GO:0003779	actin binding	14	14/355	444/28438	0.0015169	0.0345668
GO:0001614	purinergic nucleotide receptor activity	3	3/355	19/28438	0.0016112	0.0345668
GO:0001846	opsonin binding	3	3/355	19/28438	0.0016112	0.0345668

(C) Gene Ontology enrichment table - Cellular Component (CC)

ID	Description	Count	GeneRatio	BgRatio	pvalue	qvalue
GO:0042613	MHC class II protein complex	6	6/361	12/28886	0.0000000	0.0000008
GO:0045121	membrane raft	19	19/361	392/28886	0.0000006	0.0000526
GO:0098857	membrane microdomain	19	19/361	393/28886	0.0000006	0.0000526
GO:0005925	focal adhesion	12	12/361	169/28886	0.0000016	0.0000969

GO:0005681	spliceosomal complex	13	13/361	206/28886	0.0000022	0.0001075
GO:0030055	cell-substrate junction	12	12/361	181/28886	0.0000033	0.0001323
GO:0043235	receptor complex	18	18/361	408/28886	0.0000046	0.0001604
GO:0042611	MHC protein complex	6	6/361	38/28886	0.0000072	0.0002198
GO:0030532	small nuclear ribonucleoprotein complex	7	7/361	66/28886	0.0000186	0.0005040
GO:0071004	U2-type prespliceosome	4	4/361	15/28886	0.0000294	0.0006520
GO:0071010	prespliceosome	4	4/361	15/28886	0.0000294	0.0006520
GO:0120114	Sm-like protein family complex	7	7/361	78/28886	0.0000554	0.0010891
GO:0005765	lysosomal membrane	10	10/361	171/28886	0.0000624	0.0010891
GO:0098852	lytic vacuole membrane	10	10/361	171/28886	0.0000624	0.0010891
GO:0097525	spliceosomal snRNP complex	6	6/361	58/28886	0.0000855	0.0013928
GO:0005795	Golgi stack	7	7/361	86/28886	0.0001035	0.0015802
GO:0031985	Golgi cisterna	6	6/361	61/28886	0.0001137	0.0016337
GO:0005774	vacuolar membrane	11	11/361	228/28886	0.0001521	0.0020641
GO:0005684	U2-type spliceosomal complex	7	7/361	95/28886	0.0001935	0.0024867
GO:0032432	actin filament bundle	7	7/361	97/28886	0.0002202	0.0026885
